# Anthropometric variables as predictors of aspects of quality of life in persons with central obesity

**DOI:** 10.1186/s13104-018-3787-6

**Published:** 2018-09-25

**Authors:** Shirley Telles, Niranjan Kala, Sachin Kumar Sharma, Acharya Balkrishna

**Affiliations:** Patanjali Research Foundation, Patanjali Yogpeeth, Haridwar, Uttarakhand 249405 India

**Keywords:** Central obesity, Anthropometry, Indian adults, Quality of life

## Abstract

**Objective:**

Central obesity has been shown to negatively influence the quality of life in centrally obese persons of both sexes. In a population of 740 centrally obese Asian-Indian adults, the present study was conducted to determine whether body mass index (BMI), waist circumference, hip circumference (HC), waist-hip ratio (WHR) and sagittal abdominal diameter (SAD) could predict different domains of quality of life. The differences based on gender and age were also determined. Linear regression analyses were carried out and the level of statistical significance (α) was set at 0.05.

**Results:**

Body mass index, HC, WHR and SAD were significant predictors for different domains of quality of life as well as for the summated total quality of life. BMI was found to be the most important predictor among all predictors across age groups and both sexes.

## Introduction

Asian Indians show relatively greater accumulation of visceral fat and susceptibility to central obesity and this has been termed as the “Asian Indian phenotype” [1, 2]. An ongoing ICMR-INDIAB study surveyed 16,000 individuals aged 20 years and above from urban as well as rural areas of India [3]. The first phase of the study reported a higher prevalence of isolated central obesity compared to generalized obesity independent of ethnicity. Central obesity is worrisome considering its association with an increased risk of endothelial dysfunction, diabetes mellitus, cardiovascular disease, inflammation, insulin resistance, metabolic syndrome, hypercholesterolemia, and certain cancers [4].

Apart from the risks of developing specific disorders, central obesity has been shown to negatively influence other aspects of life. In 56 overweight and obese Brazilian women with a short stature who had central obesity there was a tendency towards low self-esteem and a negative body image [5]. In another study on 246 Iranian soldiers, an inverse association was reported between self-esteem and central obesity [6]. Central obesity also influenced work ability in 11,637 Finnish persons who showed an association between the waist-hip ratio (WHR) and low work ability index [7]. In males especially, abdominal obesity has been associated with lower testosterone levels and libido [8, 9]. Some of these effects are due to chemicals secreted by the adipose tissue deposited intra-peritoneally, while other effects are more a product of social and psychological factors which can influence the mood.

The present cross-sectional study aimed at (i) determining whether the body mass index, waist circumference, hip circumference, waist-hip ratio and sagittal abdominal diameter could predict the quality of life in 740 persons with central obesity and (ii) to determine differences based on gender and age.

## Main text

### Methods

#### Study design and recruitment of research participants

The study was a cross sectional trial. Seven hundred and forty centrally obese persons of both sexes were recruited from four regions of India (419 females; group mean age ± SD, 44.9 ± 10.5 years). The participants were from north (34.5%), south (34.2%), east (13.9%) and west (17.4%) regions of India based on a standard categorization of the regions [10]. The inclusion criteria were (i) presence of central obesity i.e., waist circumference ≥ 80 cm for women and ≥ 90 cm for men [11], (ii) not currently taking part in any weight loss program and (iii) willingness to take part in the study. The criteria for exclusion were (i) presence of diseases/conditions associated with obesity such as type 2 diabetes mellitus and cardiovascular disease and (ii) obesity secondary to hormonal imbalance, other medical conditions or medication such as steroids. Participants were recruited through advertisements in local newspapers and flyers with the help of social workers connected to the research institute conducting the research. The participants’ signed informed consent was taken individually. Ethical approval from the Ethics Committee of Patanjali Research Foundation, Haridwar, India was obtained (Approval No. YRD-017/022).

#### Assessments

The assessments are mentioned below.

#### Waist circumference (WC)

Participants were lightly clothed and asked to stand upright with their weight evenly distributed on both feet. The tape measure (Gülick Anthropometric tape Model J00305, Lafayette Instrument, U.S.A.) was wrapped around the abdomen between the iliac crest and the inferior costal margin. The tape was kept parallel to the floor and the measurement was taken to the nearest 0.1 cm after a normal exhalation.

#### Sagittal abdominal diameter (SAD)

The participants were asked to lie supine on their back. A caliper was used with two sliding arms attached parallel to a vertical scale [Holtain-Kahn Abdominal Caliper 50 cm (98.609XL), U.K.] following the standard method for the use of the caliper [12]. The lower arm of the caliper was placed at the level of L4–L5 under the participant and the upper arm of the caliper was lowered without compressing the abdomen. The reading on the vertical scale after a normal exhalation was noted in cm.

#### Hip circumference (HC)

The hip circumference was measured around the pelvis at the point of maximal protrusion of the buttocks in a horizontal plane.

#### Body mass index (BMI)

The body mass index (BMI) was calculated as the body weight (in kg), divided by the height (in meters) squared.

#### Waist-hip ratio (WHR)

The ratio of the waist circumference to the hip circumference was derived as WC (in centimeters) divided by HC (in centimeters) or the waist-hip ratio (WHR).

#### Quality of life (QoL)

The quality of life was assessed using Moorehead-Ardelt quality of life (QoL) Questionnaire which consists of six items that assess six different aspects of quality of life viz., general self-esteem, physical activity, social contacts, satisfaction concerning work, pleasure related to sexuality, and focus on eating behavior [13]. The scores range from − 0.5 to 0.5. The sum of these 6 scores provides an overall QoL score. Each score is classified into five categories (very poor: − 3.0 to − 2.1; poor: − 2.0 to − 1.1; fair: − 1.0 to 1.0; good: 1.1 to 2.0: and very good: 2.1 to 3.0).

### Data analysis

Data were analyzed using SPSS (Version 24.0).

Separate linear regression analyses were carried out to determine if the five anthropometric variables (i.e., BMI, WC, HC, WHR, and SAD) would predict six domains of quality of life as well as total quality of life based on anthropometric measures, in a regression analysis model. The data were divided gender-wise (as 321 males and 419 females) and age-wise as three age groups i.e., 18–30 years, 31–50 years and 51–70 years. It has been reported that age affects various aspects of quality of life differently in overweight and obese persons [14]. For example those aspects of quality of life which involve physical functions deteriorate with increasing age in overweight and obese persons whereas self-esteem and public distress improve with age. Hence in the present study age wise categorization was done. Separate linear regression analyses were further carried out on these groups to see the age and gender specific variations in the results.

### Results

Results of the of linear regression analyses for the whole group, both genders and three age groups are given in Table 1.

**Table 1:** Results of linear regression analyses for the whole group, both genders and three age groups

	Predicting variable	Outcome variable	Adjusted R^2^	Beta coefficient	CI		P value
					Upper	Lower	
Whole group (n = 740; m:f = 321:419)	BMI	Pleasure related to sexuality	0.014	− 0.125	− 0.003	− 0.012	0.001
		Total quality of life	0.008	− 0.098	− 0.006	− 0.038	0.007
	WHR	Pleasure related to sexuality	0.005	0.078	0.427	0.018	0.033
	HC	Pleasure related to sexuality	0.011	− 0.111	− 0.001	− 0.005	0.002
	SAD	Total quality of life	0.006	− 0.084	− 0.002	− 0.031	0.023
Females (n = 419)	BMI	Social satisfaction	0.013	− 0.123	− 0.001	− 0.011	0.012
		Ability to work	0.008	− 0.104	0.000	− 0.010	0.033
		Pleasure related to sexuality	0.007	− 0.098	0.000	− 0.012	0.046
		Total quality of life	0.015	− 0.133	− 0.008	− 0.050	0.007
Males (n = 321)	SAD	Total quality of life	0.010	− 0.117	− 0.002	− 0.061	0.037
Age 18–30 (n = 82; m:f = 38:44)	BMI	General self esteem	0.045	− 0.239	− 0.001	− 0.023	0.031
		Pleasure related to sexuality	0.036	− 0.219	0.000	− 0.022	0.048
Age 31–50 (n = 429; m:f = 175:254)	BMI	Pleasure related to sexuality	0.010	− 0.109	− 0.001	− 0.012	0.024
	HC	Pleasure related to sexuality	0.011	− 0.114	− 0.001	− 0.006	0.018
Age 51–70 (n = 229; m:f = 108:121)	BMI	General self esteem	0.016	− 0.142	− 0.001	− 0.015	0.031
		Ability to work	0.038	− 0.206	− 0.005	− 0.021	0.002
		Social satisfaction	0.024	− 0.169	− 0.002	− 0.015	0.010
		Pleasure related to sexuality	0.017	− 0.146	− 0.001	− 0.019	0.027
		Focus on eating behavior	0.016	− 0.143	− 0.001	− 0.016	0.031
		Total quality of life	0.054	− 0.241	− 0.028	− 0.090	< 0.001
	HC	Pleasure related to sexuality	0.013	− 0.132	0.000	− 0.007	0.045
		Total quality of life	0.021	− 0.158	− 0.003	− 0.029	0.016

*CI* confidence interval, BMI: body mass index, *HC* hip circumference, *m:f* male:female ratio, *SAD* sagittal abdominal diameter, *WHR* waist hip ratio

#### Regression analyses for the whole group (n = 740)

In the whole group, BMI acted as a significant negative predictor for total quality of life (beta coefficient = − 0.098, P = 0.007) and for pleasure related to sexuality (beta coefficient = − 0.125, P = 0.001). WHR positively predicted pleasure related to sexuality (beta coefficient = 0.078, P = 0.033), HC negatively predicted pleasure related to sexuality (beta coefficient = − 0.111, P = 0.002) and SAD negatively predicted total quality of life (beta coefficient = − 0.184, P = 0.023) in this group.

#### Regression analysis gender-wise

##### Regression analyses for females (n = 419)

BMI was found to be a significant negative predictor for social satisfaction (beta coefficient = − 0.123, P = 0.012), ability to work (beta coefficient = − 0.104, P = 0.033), pleasure related to sexuality (beta coefficient = − 0.198, P = 0.046) and total quality of life (beta coefficient = − 0.133, P = 0.007) in females.

##### Regression analyses for males (n = 321)

SAD negatively predicted total quality of life (beta coefficient = − 0.117, P = 0.037) in males.

#### Regression analysis age-wise

##### Regression analyses for age group 18–30 (n = 82)

BMI negatively predicted general self esteem (beta coefficient = − 0.239, P = 0.031) and pleasure related to sexuality (beta coefficient = − 0.219, P = 0.048) in this age group.

##### Regression analyses for age group 31–50 (n = 429)

BMI negatively predicted pleasure related to sexuality (beta coefficient = − 0.109, P = 0.024) and HC also negatively predicted pleasure related to sexuality (beta coefficient = − 0.114, P = 0.018) in this age group.

##### Regression analyses for age group 51–70 (n = 70)

In this age group, BMI negatively predicted general self esteem (beta coefficient = − 0.142, P = 0.031), ability to work (beta coefficient = − 0.206, P = 0.002), social satisfaction (beta coefficient = − 0.169, P = 0.010), pleasure related to sexuality (beta coefficient = − 0.146, P = 0.027), focus on eating behavior (beta coefficient = − 0.143, P = 0.031) and total quality of life (beta coefficient = − 0.241, P < 0.001) significantly. HC negatively predicted pleasure related to sexuality (beta coefficient = − 0.132, P = 0.045) and total quality of life (beta coefficient = − 0.158, P = 0.016) in this age group.

## Discussion

In 740 adult Indians with central obesity, several anthropometric measures predicted the quality of life. The BMI was the most important predictor of different aspects of quality of life, which was true across age groups and gender.

The total quality of life (QoL) was negatively predicted by three variables, BMI, SAD and HC. The BMI predicted the total QoL in the whole group, females and old persons (i.e., aged 51–70 years). The sagittal abdominal diameter (SAD) predicted total QoL in the whole group and in all males. The SAD is a better measure of visceral fat rather than subcutaneous fat [12]. Visceral fat contributes to chronic inflammation which is associated with various diseases including cancer [15]. The association between high SAD with poor QoL could be related to these negative effects on health.

The HC negatively predicted the total Qol in old persons. In a Swedish female cohort, larger hip circumference was associated with significantly fewer adverse health outcomes [16]. However this relation seems to be ethnicity dependent, since in an Asian-Chinese population larger hip circumference was associated with incident high triglycerides, low HDL-C, and multiple metabolic abnormalities [17]. In the absence of data on an Asian-Indian population, the data on Asian-Chinese persons may be considered of greater relevance to the present study, despite the considerable genetic diversity of Asian-Indians [18]. Hence the HC is also an independent predictor of health risks which could explain its relation with the total QoL.

The BMI acted as a negative predictor for general self esteem in persons of a young age group (i.e., 20–30 years) as well as in an old age group (i.e., 51–70 years). Irrespective of physical characteristics, self esteem is believed to have a trajectory across the course of life, increasing during young and middle adulthood, reaching a peak at about 60 years, and then declining in old age [19]. This could contribute to the negative association between BMI and self esteem in old age. In the younger age group, obesity is associated with psychosocial consequences such as perceived stress and social marginalization [20, 21], which could contribute to the relation found between low self esteem and higher BMI.

The BMI was a negative predictor for satisfactory social contacts in females as well as in old persons. A direct association between BMI and depressive symptoms has been reported along with influencing the body image [22]. The association was gender dependent with a greater effect on females than in males. Mental depression along with poor body image can be responsible for unsatisfactory social contacts in females and old persons with a high BMI.

The ability to work was negatively predicted by the BMI in females and old persons. This could be explained by earlier studies reporting increased neuromuscular fatigue related to stress, a sedentary lifestyle and obesity in both men and women [23, 24]. Work ability was previously shown to be inversely related to the BMI in a Finnish population [7].

Pleasure related to sexuality was predicted by three variables, BMI, HC and WHR; with BMI being a significant negative predictor for all cohorts except males. HC was a negative predictor in the whole group, middle aged persons (i.e., 31–50 years) and old persons (i.e., 51–70 years), while WHR was a positive predictor in the whole group. Hip circumference was negatively correlated with sperm concentration in males [25]. Low testosterone levels and decreased libido were associated with obesity and metabolic syndrome [8, 9]. Hence BMI and HC appear to predict impaired reproductive health, which could influence the aspect of quality of life related to sexuality. The reason why the waist-hip ratio could be positively related to sexual health is the negative relation with the HC (the denominator of the ratio).

The approach towards food in older persons (i.e., 51–70 years) was negatively predicted by the BMI in our study. The reason could be that older people with a high BMI are concerned about their health and food choices [26].

In summary in the population of centrally obese adult Asian Indians given the genetic predisposition to central obesity, anthropometric indices studied, viz. BMI, SAD, HC and WHR acted as significant predictors of quality of life, though the BMI was the best predictor across age groups and for both sexes.

## Limitations

In the present cross-sectional study external validity may be compromised as the results may be applicable mainly to the population studied (i.e., Asian Indians with central obesity) but may not be generalizable to a wider population (for example, to all centrally obese populations world wide or to Asian Indians with overall obesity rather than central obesity alone) [27]. This is a limitation of the study.

## Abbreviations

BMI: body mass index
CI: confidence interval
HC: hip circumference
HDL-C: high density lipoprotein cholesterol
ICMR-INDIAB: Indian Council of Medical Research-India Diabetes
QoL: quality of life
SAD: sagittal abdominal diameter
SD: standard deviation
SPSS: Statistical Package for Social Sciences
WC: waist circumference
WHR: waist-hip ratio
